# Genotyping and drug susceptibility testing of mycobacterial isolates from population-based tuberculosis prevalence survey in Ghana

**DOI:** 10.1186/s12879-017-2853-3

**Published:** 2017-12-02

**Authors:** Kennedy Kwasi Addo, Samuel Ofori Addo, Gloria Ivy Mensah, Lydia Mosi, Frank Adae Bonsu

**Affiliations:** 1 Department of Bacteriology, Noguchi Memorial Institute for Medical Research, University of Ghana, P.O. Box LG 581, Legon, Ghana; 2 West African Center for Cell Biology of Infectious Pathogens, University of Ghana, Legon, Ghana; 3 Department of Biochemistry, Cell and Molecular Biology, University of Ghana, Legon, Ghana; 4 National Tuberculosis Programme, Ghana Health Service, Accra, Ghana

**Keywords:** Tuberculosis, Prevalence survey, Ghana, Line probe assay, Drug resistance, MTBDR*plus*, NTM-DR

## Abstract

**Background:**

*Mycobacterium tuberculosis* complex (MTBC) and Non-tuberculosis Mycobacterium (NTM) infections differ clinically, making rapid identification and drug susceptibility testing (DST) very critical for infection control and drug therapy. This study aims to use World Health Organization (WHO) approved line probe assay (LPA) to differentiate mycobacterial isolates obtained from tuberculosis (TB) prevalence survey in Ghana and to determine their drug resistance patterns.

**Methods:**

A retrospective study was conducted whereby a total of 361 mycobacterial isolates were differentiated and their drug resistance patterns determined using GenoType Mycobacterium Assays: MTBC and CM/AS for differentiating MTBC and NTM as well MTBDR*plus* and NTM-DR for DST of MTBC and NTM respectively.

**Results:**

Out of 361 isolates, 165 (45.7%) MTBC and 120 (33.2%) NTM (made up of 14 different species) were identified to the species levels whiles 76 (21.1%) could not be completely identified. The MTBC comprised 161 (97.6%) *Mycobacterium tuberculosis* and 4 (2.4%) *Mycobacterium africanum*. Isoniazid and rifampicin monoresistant MTBC isolates were 18/165 (10.9%) and 2/165(1.2%) respectively whiles 11/165 (6.7%) were resistant to both drugs. Majority 42/120 (35%) of NTM were *M. fortuitum*. DST of 28 *M. avium* complex and 8 *M. abscessus complex* species revealed that all were susceptible to macrolides (clarithromycin, azithromycin) and aminoglycosides (kanamycin, amikacin, and gentamicin).

**Conclusion:**

Our research signifies an important contribution to TB control in terms of knowledge of the types of mycobacterium species circulating and their drug resistance patterns in Ghana.

**Electronic supplementary material:**

The online version of this article (10.1186/s12879-017-2853-3) contains supplementary material, which is available to authorized users.

## Background

Sub-Saharan Africa accounted for about 28% of the estimated 9.6 million of all notified tuberculosis (TB) cases in 2014. The TB menace is further aggravated by the emergence of drug resistant strains which is a setback in TB control efforts. In Ghana, TB still poses a major public health concern with a total of 14,668 cases reported in 2014 [1]. The Ghana National TB prevalence survey was conducted from March – December, 2013 to obtain useful estimates of TB prevalence within the time period. The adjusted prevalence of smear positive pulmonary TB and bacteriologically confirmed TB among adults aged 15 years and above were 111 (95% CI 76–145) per 100,000 population and 356 (95% CI 288–425) per 100,000 population respectively (personal communication with the programme manager for the National TB Control Programme, Ghana, unpublished data). Isolation of many Nontuberculous *Mycobacterium* (NTM) during the survey necessitated further investigations. *Mycobacterium tuberculosis* complex (MTBC) and Non-tuberculosis *Mycobacterium* (NTM) infections differ clinically, making rapid identification and drug susceptibility testing (DST) very critical for infection control and drug therapy [2]. The use of simple, rapid and efficient tools for mycobacterial differentiation and DST such as World Health Organization (WHO) endorsed line probe assay (LPA) is much needed especially in developing countries with high TB burden and preponderance of environmental mycobacteria [3]. One of such commercially available LPA is the GenoType Mycobacterium Assays (Hain Lifescience, Nehren, Germany) which have demonstrated high sensitivity and specificity in many studies for differentiating between species of MTBC and NTM as well as determining the presence of mutations in genes associated with drug resistance [4–6]. This study therefore sought to use LPA to differentiate mycobacterial isolates and thereafter determine their resistance to anti-mycobacterial drugs. To the best of our knowledge this is the first study in Ghana to genotype and determine DST patterns of both MTBC and NTM strains obtained from a nationwide TB prevalence survey.

## Methods

### Study design and population

This study was a follow-up to the National TB prevalence survey conducted from March–December, 2013 to determine the actual burden of TB in adult population (≥ 15 years) in Ghana. The survey was cross sectional and community-based in 98 clusters in two strata (urban, rural) which were selected by stratified multistage cluster sampling based on probability proportional to size. All participants were screened using an interview about symptoms and chest X-ray as recommended by WHO [7]. A total of 8298 participants were eligible for sputum examination, of whom 8126 (98%) submitted at least one sputum specimen and 7706 (93%) submitted two sputum specimens. This current study was done using culture positive mycobacterial isolates identified from the prevalence survey. Additional file 1 represents the map of Ghana showing the study sites (clusters).

### Laboratory procedures

All laboratory investigations were carried out in a Pathogen Level 3 (P3) Laboratory.

#### Culture and identification

Equal volumes of commercially available Sodium hydroxide-N-acetyl L-cysteine (NaOH-NALC)-Mycoprep (BD Diagnostic System, Sparks, MD, USA) was added to sputum in a 50 ml centrifuge tube. The mixture was vortexed and allowed to stand for 15 min at room temperature. Phosphate buffer saline (PBS) [pH = 6.8] was added up to the 50 ml mark and centrifuged at 3000 g at 4 °C for 15 min. The supernatant was discarded to obtain sediment which was reconstituted with 2 ml of PBS (pH = 6.8). An aliquot (0.5 ml) of reconstituted sediment was inoculated into two tubes of Mycobacteria Growth Indicator Tube (MGIT) and incubated in the BACTEC MGIT 960 (BD Diagnostic System, Sparks, MD, USA) for a maximum of 42 days. Once a tube flagged positive in MGIT, smear was prepared and Ziehl Neelsen (ZN) stained for the presence or absence of acid fast bacilli (AFB). Additionally, a portion of the positive culture was inoculated onto blood agar plates by streaking to check for contamination. All the positive mycobacterial isolates obtained were broadly identified as *Mycobacterium tuberculosis* complex (MTBC) and Non-tuberculosis mycobacterium (NTM) using BD MGIT™ TBc Identification Test Kit (BD Diagnostic System, Sparks, MD). The test detects the MPT64 antigen which is highly specific for MTBC. The test and interpretation of the results was done according to manufacturer’s instructions [8]. All negative test samples were suspected NTM and confirmed using GenoType Mycobacterium CM and GenoType Mycobacterium AS (Hain Lifescience Nehren, Germany).

### Line Probe Assay (LPA)

The LPA procedure consisting of DNA extraction, master mix preparation, polymerase chain reaction (PCR) and reverse hybridization were performed in separate rooms. All the assays (GenoType MTBC; GenoType Mycobacterium CM; GenoType Mycobacterium AS; GenoType MTBDR*plus*, GenoType NTM-DR) were run according to manufacturer’s instructions [9–13]. Quality control of all the tests were ensured by using H37Rv strain and nuclease free water as positive and negative control markers respectively.

### DNA extraction

The boiling (heat killing) method of DNA extraction was used. About 0.5 ml of thawed suspension of isolates was dispensed into a 1.5 ml screw capped micro centrifuge tubes. The tubes were placed in a heating block at 90 °C for about 1 h to disrupt the cell wall and release DNA into solution. The solution was allowed to stand for about 15 min without any form of shaking in order that the disrupted cells will settle down in the tubes. Next, the supernatant containing the mycobacterial DNA was carefully transferred into different tubes using Pasteur pippette and kept at −20 °C until used for PCR.

### Multiplex amplification with biotinylated primers

Briefly, 10 μl of Amplification Mix A (AM-A) consisting of 5 μl 10× buffer, 2 μl MgCl_2_, 3 μl of molecular grade water and 0.2 μl (1 U) Taq DNA polymerase was mixed with 35 μl of Amplification Mix B (AM-B) made up of nucleotides, biotinylated primers and dye. Then, 5 μl of extracted DNA sample was added to the master-mix in another room. PCR amplification was done with the thermocycler set at the following cycling conditions: one cycle at 95 °C for 15 min, followed by ten cycles at 95 °C and 58 °C for 30 s and 2 min respectively. This was followed by another round of 20 cycles at 95 °C, 53 °C and 70 °C for 25, 40 and 40 s respectively before a final single cycle at 70 °C for 8 min.

### Reverse hybridization

Hybridization was carried out with the GT-Blot 48® automated hybridizer (Hain Lifescience Nehren, Germany) in accordance with manufacturer’s instructions. Pre-heated hybridization and stringent buffers, diluted conjugate and substrate solutions, rinsing and sterile distilled water were placed into their respective colour-coded slots in the GT-Blot 48®. Then suction heads were placed into corresponding colour-coded solutions. Equal volumes (20 μl) of denaturing reagent and amplicons were mixed together in wells of a tray placed in the GT-Blot 48® and allowed to stand for 5 min at 25 °C. After denaturation, the test strips with sample identification numbers written on them were placed into corresponding wells. The automated process of dispensing and aspirating various solutions was set in a sequential order: hybridization, stringent wash, rinsing, conjugate, rinsing, sterile distilled water, substrate, sterile distilled water. The strips were then dried using the heating systems in the GT-Blot 48®.

### Evaluation and interpretation of results

The fully dried strips were scanned using GenoScan® (Hain Lifescience, Nehren, Germany) which generated an automated read-out of the band patterns. The strips were pasted on evaluation sheets included in the kit. The final results on the read-out were verified manually with the naked eye.

### Statistical analysis

All the data collected were entered into Microsoft Excel 2013 (Microsoft Corporation, USA) for analysis. Results were presented in tables and graphs showing frequencies and percentages. Fisher’s exact test was applied to determine association between socio-demographic characteristics, with *p* ≤ 0.05 considered statistically significant.

### Ethical considerations

The main study (Assessing tuberculosis disease prevalence in Ghana through a population based survey) obtained ethical approval from the Institutional Review Board (IRB) of Noguchi Memorial Institute for Medical Research (NMIMR) [FWA 00001824; IRB 00001276].

## Results

### Background characteristics

Out of 361 culture positive mycobacterial isolates, 159 (44%) and 202 (56%) were obtained from male and female participants respectively. Based on the BD MGIT™ TBc Identification Test Kit, 165 (45.7%) were identified as MTBC whiles 196 (54.3%) were suspected to be NTM. The mean age of culture positive participants were 47.4 years and 45.9 years for MTBC and NTM respectively. Background characteristics of participants from whom mycobacterial isolates were obtained have been summarized in Table 1.

**Table 1:** Characteristics of participants from whom mycobacterial isolates used in the study were obtained (*N* = 361)

Characteristic	MTBC	*p* value	NTM	*p* value	Total
	*n* (%)		*n* (%)		*N* (%)
Gender					
Male	91 (55.2)	0.0001	68 (34.7)		159 (44.0)
Female	74 (44.8)		128 (65.3)	0.0001	202 (56.0)
Age group					
15–24	25 (15.2)		33 (16.8)		58 (16.1)
25–34	22 (13.3)		35 (17.9)		57 (15.8)
35–44	22 (13.3)		31 (15.8)		53 (14.7)
45–54	31 (18.8)		22 (11.2)		53 (14.7)
55–64	23 (13.9)		20 (10.2)		43 (11.9)
65+	42 (25.5)	0.63	55 (28.1)	0.63	97 (26.8)
TB History^a^					
Yes	6 (3.6)		0 (0)		6 (1.7)
No	159 (96.4)		196 (100)		355(98.3)

*MTBC Mycobacterium tuberculosis* complex, *NTM* Non-tuberculous mycobacterium

^a^Participants who have been diagnosed with TB and treated prior to the prevalence survey

### Differentiation of MTBC (GenoType MTBC)

Out of 165 MTBC isolates differentiated, 161 (97.6%) were identified as *M. tuberculosis* while 4 (2.4%) were *M. africanum*. Other members of MTBC such as *M. bovis* and *M. microti* that has been reported to cause disease in humans were not identified (Table 2).

**Table 2:** Frequency of MTBC species differentiated by GenoType MTBC Assay (*N* = 165)

MTBC species	Number	Percentage (%)
*M. tuberculosis*	161	97.6
*M. africanum*	4	2.4
*M. bovis*	0	0.0
*M. microti*	0	0.0

*MTBC Mycobacterium tuberculosis* complex, *M. tuberculosis Mycobacterium tuberculosis*, *M. africanum Mycobacterium africanum*, *M. bovis Mycobacterium bovis*, *M. microti Mycobacterium microti*

### Drug susceptibility testing of MTBC (GenoType MTBDR*plus*)

**Fig. 1:**
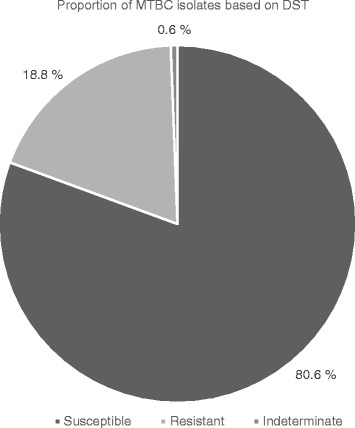
Drug resistance patterns of MTBC isolates (*N* = 165). Susceptible: isolates that are susceptible to both isoniazid and rifampicin. Resistant: isolates that are resistant to at least isoniazid and rifampicin. Indeterminate: isolate whose resistance profile to isoniazid or rifampicin could not be determined by the GenoType MTBDR*plus*

Out of 165 MTBC isolates, 133 (80.6%) were susceptible to both isoniazid (INH) and rifampicin (RIF), 31 (18.8%) were resistant to either INH or RIF while the DST status of the remaining 1 (0.6%) could not be determined (assay yielded an indeterminate result) (Fig. 1). The resistance pattern was 18/165 (10.9%), 2/165 (1.2%) and 11/165 (6.7%) for INH monoresistance, RIF monoresistance and multi-drug resistance-MDR (resistant to both INH and RIF) respectively. One out of two RIF monoresistant isolates had mutations in codon 530–533 resulting in the absence of wild type band (WT8) without the presence of the corresponding mutation band whiles the other showed no mutation in the wild type but MUT 3 (S531 L) mutation (Table 3). For INH monoresistant isolates, mutations in *katG* MUT1 (S315 T1)-only was the most frequent, making up 14/18 (77.8%) while the remaining 4/18 (22.2%) had mutations in *inhA* MUT1 (C15T) with the absence of corresponding WT1 (−15/−16). None of the INH monoresistant isolates had mutations in both *katG* and *inhA*. The most frequent mutations found in MDR isolates were D516V [6/11 (54.5%)], S315 T1 [10/11 (90.9%)] and T8C [6/11 (54.5%)] in the *rpoB*, *katG* and *inhA* respectively. Other mutations in *rpoB* included S531 L 1/11 (9.1%) and H526Y 3/11(27.3%) (Table 3).

**Table 3:** Specific gene mutations in resistant strains

Gene locus	Band	Gene region/mutation	^a^RIF monoresistant	^b^INH monoresistant	^c^MDR
*rpoB*					
	WT1	506–509			
	WT2	510–513			
	WT3	513–517			^d^1
	WT4	516–519			
	WT5	518–522			
	WT6	521–525			
	WT7	526–529			^d^3
	WT8	530–533	1		1
	MUT1	D516V			6
	MUT2A	H526Y			^d^3
	MUT2B	H526D			
	MUT3	S531 L	1		1
*katG*					
	WT	315		^d^11	^d^4
	MUT1	S315 T1		14	10
	MUT2	S315 T2			
*inhA*					
	WT1	−15/−16		^d^4	^d^1
	WT2	−8			^d^1
	MUT1	C15T		^d^4	^d^1
	MUT2	A16G			
	MUT3A	T8C			6
	MUT3B	T8A			

*WT* wild type band, *MUT* mutant band, *RIF* rifampicin, *INH* isoniazid, *MDR* multi-drug resistance

^a^Isolates with mutation(s) in the rpoB gene and none in the inhApro or katG gene

^b^Isolates with mutation(s) in the inhApro region and/or in the katG gene, with no mutation in the rpoB gene

^c^Isolates with mutations in the rpoB gene and inhApro and/or katG gene

^d^Number of isolates that had presence of mutation band and the absence of corresponding wild type band

### Differentiation of NTM (GenoType Mycobacterium CM/GenoType Mycobacterium AS)

**Fig. 2:**
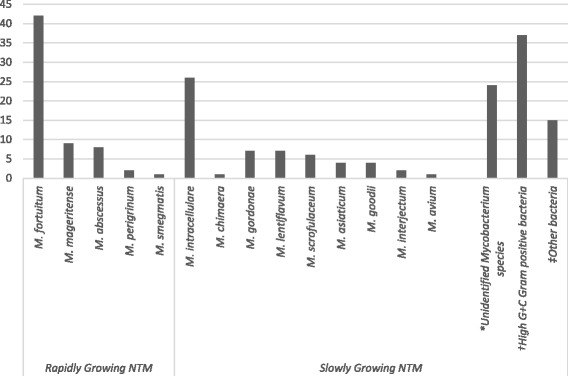
Identification of species among suspected NTM isolates. *M. fortuitum* = *Mycobacterium fortuitum*; *M. mageritense* = *Mycobacterium mageritense; M. abscessus* = *Mycobacterium abscessus*; *M. perigrinum* = *Mycobacterium perigrinum; M. smegmatis* = *Mycobacterium smegmatis*; *M. intracellulare* = *Mycobacterium intracellulare; M. chimaera* = *Mycobacterium chimaera*; *M. gordonae* = *Mycobacterium gordonae; M. lentiflavum* = *Mycobacterium lentiflavum*; *M. scrofulaceum* = *Mycobacterium scrofulaceum; M. asiaticum* = *Mycobacterium asiaticum*; *M. goodii* = *Mycobacterium goodii; M. interjectum* = *Mycobacterium interjectum*; *M. avium* = *Mycobacterium avium*; N = number of isolates tested. *Mycobacterium species that could not be identified to the species level. †Members of a group of gram-positive bacterium with high guanine and cytosine content. ‡Group of bacteria that were neither mycobacteria nor high G + C gram-positive bacteria according to assay kit manufacturer’s instructions

Out of 196 isolates suspected to be NTM, 120 (61.2%) were identified completely to the species level. Among these were *M. fortuitum* 42 (21.4%), *M. intracellulare/ M. chimaera* 27 (13.8%), *M. mageritense* 9 (4.6%), *M. abscessus* 8 (4.1%), *M. gordonae* 7 (3.6%), *M. lentiflavum* 7 (3.6%), *M. scrofulaceum* 6 (3.1%), *M. asiaticum* 4 (2.0%), *M. goodii* 4 (2.0%), *M. interjectum* 2 (1.0%), *M. perigrinum* 2 (1.0%), *M. avium* 1 (0.5%) and *M. smegmatis* 1 (0.5%). The remaining 76 (38.8%) could not be identified completely using both GenoType CM and GenoType AS. Based on individual band pattern and the use of interpretation chart from the kit manufacturer, these isolates were only identified as *Mycobacterium* species 24 (12.2%) and High G + C Gram positive bacterium 37 (18.9%) whiles the remaining 15 (7.7%) did not correspond to any of the band patterns described in the interpretation chart (Fig. 2).

### Drug susceptibility testing of NTM (GenoType NTM-DR)

Thirty six NTM isolates comprising *M. avium* complex (*M. avium*-1; *M. intracellulare/ M.chimaera*-27) and *M. abscessus* complex (*M. abscessus* subsp. *abscessus* − 2; *M. abscessus* subsp. *massiliense*-6) species were susceptible to both macrolides (clarithromycin, azithromycin) and aminoglycosides (kanamycin, amikacin, gentamicin) when DST was performed (Table 4).

**Table 4:** Gene mutations in NTM species

Species/Subspecies	erm(41)^a^		rrl^d^		rrs^e^	
	C28^b^	T28^c^	WT	MUT	WT	MUT
*M. avium*			1		1	
*M.intracellulare*			26		26	
*M. chimaera*			1		1	
*M. abscessus* subsp. *abscessus*	2		2		2	
*M. abscessus* subsp. *massiliense*	6		6		6	
*M. abscessus* subsp. *bolletti*	0	0	0	0	0	0

*Note*: The probes erm(41) C28 and erm(41) T28 are only relevant for *M. abscessus* subsp. *abscessus* and *M. abscessus* subsp. *bolletii*, but not for *M. abscessus* subsp. *massiliense*. Due to deletions in the erm(41) gene of *M. abscessus* subsp. *massiliense* the gene is nonfunctional, leading to macrolide sensitivity in spite of a developed erm(41) T28 band (except for strains with an additional rrl mutation)

*WT* wild type probe comprises the most important resistance region of the rrl and rrs genes, *MUT* mutation probes detect the most common resistance-mediating mutations in rrl and rrs genes

^a^The erm(41) gene is examined for detection of resistance to macrolides (Clarithromycin or azithromycin) and is only present in members of the *M. abscessus* complex

^b^The erm(41) C28 probe detects a genotype that carries a C at position 28 of the erm(41) gene. When the erm(41) C28 probe stains positive, this indicates that the tested strain is sensitive to macrolides (except for strains with an additional rrl mutation)

^c^The erm(41) T28 probe detects a genotype that carries a T instead of a C at position 28 of the erm(41) gene. When the erm(41) T28 probe stains positive, this indicates that the tested strain is resistant to macrolides

^d^The rrl gene is examined for detection of resistance to macrolides (clarithromycin or azithromycin)

^e^The rrs gene is examined for detection of resistance to aminoglycosides (kanamycin, amikacin, gentamicin)

## Discussion

This study primarily set out to differentiate species of mycobacterial isolates obtained from TB prevalence survey in Ghana and to determine their resistance to some antimycobacterial drugs using commercially available LPA, the GenoType Mycobacterium Assays (Hain Life sciences, Nehren, Germany). The background data shows that culture positive MTBC isolates were higher in male participants 91 (55.2%) than females 74 (44.8%) [*p* < 0.001] which is consistent with reports from other studies [1, 14, 15]. Unlike MTBC, more isolates from females than males were identified as NTM which is consistent with report by Limo et al. [16]. A vast majority of MTBC isolates were identified as *M. tuberculosis* with very few *M. africanum*. This finding agrees with reports from many parts of the world [1, 14, 17]. The absence of *M. bovis* among isolates especially those collected from the northern and upper regions was unexpected since consumption of raw milk and pastoral activities are very common in these areas. Getahun and colleagues reported similar findings from their study in Ethiopia [18]. Determining drug resistance profile of mycobacterial isolates is very critical for effective management of the disease they cause. As expected, over 80% isolates were susceptible to both isoniazid and rifampicin because of the community-based study population from whom these isolates were obtained. Prevalence of drug resistance has been reported to be relatively low in community-based studies compared to health facility-based studies. In this study, INH monoresistance (10.9%) was relatively higher than RIF monoresistance (1.2%) which is consistent with general observation although the reverse has been reported from a study in India where out of 279 smear positive samples, 29 (10.4%) and 62 (22.2%) showed INH monoresistance and RIF monoresistance respectively [19]. Eleven out of 165 (6.7%) MTBC isolates were resistant to both INH and RIF, and thus met the definition of MDR-TB, a potential threat to public health considering the study population involved. However, taking into account the small sample size (*N* = 165) involved, this finding could not be generalized. An MDR rate of 2.2–2.5% have been reported in previous studies in Ghana [6, 20, 21]. Recently, WHO recommended that rifampicin resistant TB (RR-TB) patients should be given the same treatment regimen as MDR-TB [1]. Common mutations associated with RIF *rpoB* (D516V, H526Y and S531 L) and INH *katG* (S315 T) resistance found in our study compares with what exists in other settings [22–24]. In this study, more than half (61%) of the isolates suspected to be NTM were identified to the species level although other studies have reported as high as over 80% species level identification of NTM using GenoType Mycobacterium CM and GenoType Mycobacterium AS [25, 26]. The remaining isolates which were identified only as Mycobacteria species and High G + C Gram positive bacteria as well as those whose band pattern did not correspond to the interpretation chart could be identified through sequencing. NTM-DR determines *M. avium* complex and *M. abscessus* complex resistance to macrolides and aminoglycosides. In addition, the assay enables differentiation between *M. intracellulare* and *M. chimaera* as well as identification of subspecies of *M. abscessus.* Infections caused by these NTM are difficult to treat and the various species and subspecies within the complexes differ in drug resistance and treatment outcomes [27]. Our study had some limitations. Firstly, the lack of phenotypic testing to compare with the molecular testing used in this study. Secondly, our inability to test resistance to the other first line drugs as well as second line drugs. Lastly, though some of the most pathogenic NTM were identified by the kit, some could still not be completely identified to the species level.

## Conclusions

Our study showed that the predominant mycobacterium species causing TB in Ghana is *M. tuberculosis*. Resistance against isoniazid and rifampicin are commonly associated with mutations in the *katG* (Ser315Thr) and *rpoB* (Asp516Val) respectively. Diverse species of NTM including *M. avium* complex and *M. abscessus* complex were identified. Besides, all species and subspecies of *M. avium* complex and *M. abscessus* complex respectively were susceptible to macrolides (clarithromycin, azithromycin) and aminoglycosides (kanamycin, amikacin and gentamicin). Our research signifies an important contribution to TB control in terms of knowledge of the types of mycobacterium species circulating and drug resistance patterns in Ghana.

## Additional file

Additional file 1: Map of Ghana showing study areas. Each of the 98 clusters (activity centres) are shown in red dots across the 10 regions in Ghana. (JPEG 1202 kb)

## Abbreviations

AFB: Acid fast bacilli
AM-A: Amplification Mix A
AM-B: Amplification Mix B
DNA: Deoxyribonucleic acid
DST: Drug susceptibility testing
FWA: Federal wide assurance
INH: Isoniazid
IRB: Institutional Review Board
LPA: Line probe assay
MDR-TB: Multi-drug resistance Tuberculosis
MGIT: Mycobacterium Growth Indicator Tube
MTBC: *Mycobacterium tuberculosis* complex
NaOH-NALC: Sodium hydroxide-N-acetyl L-cysteine
NMIMR: Noguchi Memorial Institute for Medical Research
NTM: Non-tuberculosis Mycobacterium
PCR: Polymerase chain reaction
RIF: Rifampicin
RR: Rifampicin Resistant
TB: Tuberculosis
WHO: World Health Organization
ZN: Ziehl-Neelsen
