# Supplementary figures and images for: Genotyping and drug susceptibility testing of mycobacterial isolates from population-based tuberculosis prevalence survey in Ghana
Source: BMC Infect Dis. 2017 Dec 2;17:743. doi: 10.1186/s12879-017-2853-3 (PMC5712140; doi:10.1186/s12879-017-2853-3)

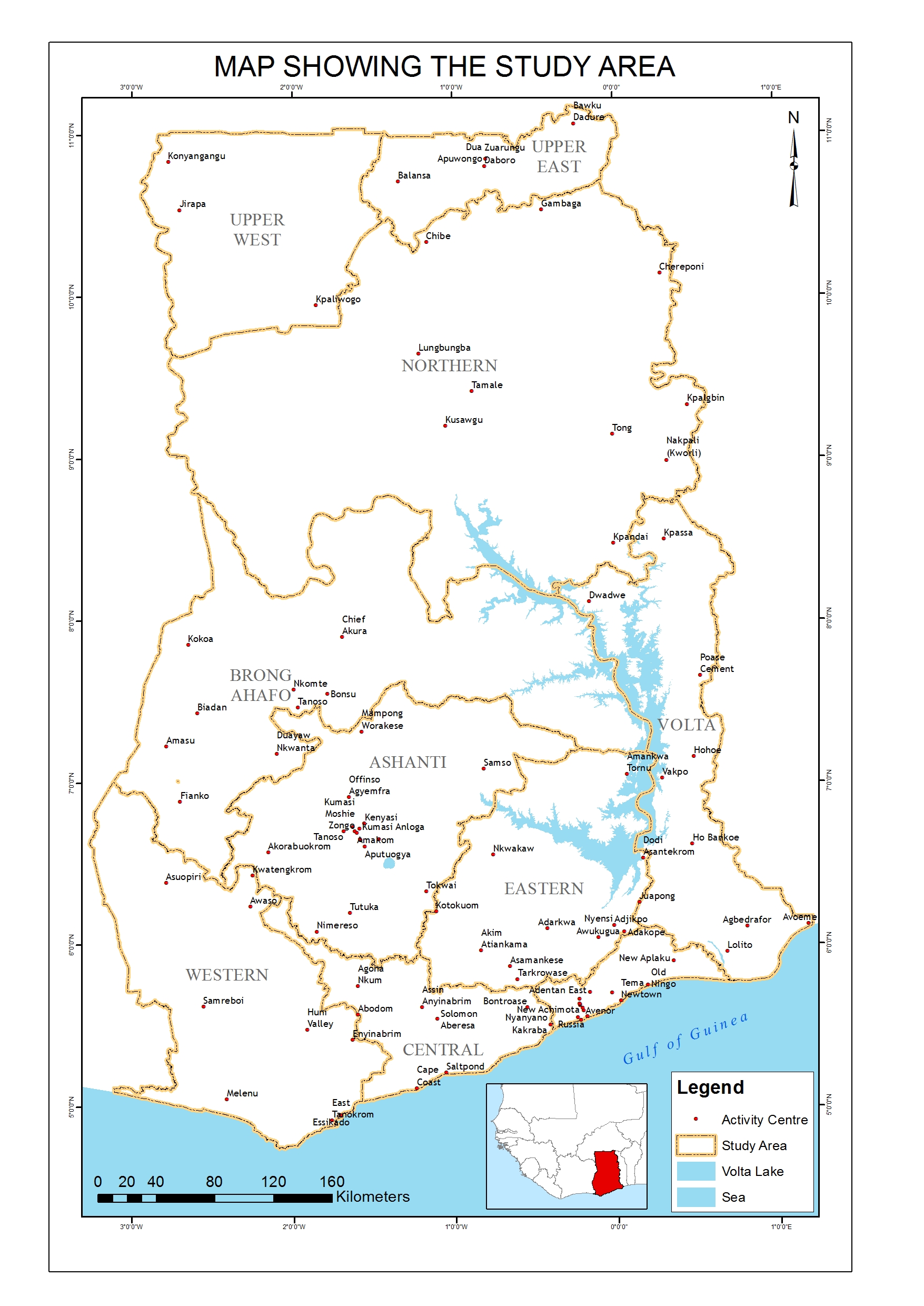

Supplement: Additional file 1: — Map of Ghana showing study areas. Each of the 98 clusters (activity centres) are shown in red dots across the 10 regions in Ghana. (JPEG 1202 kb) [file 12879_2017_2853_MOESM1_ESM.jpg]
